# Comparative Genomics of Fungi in Nectriaceae Reveals Their Environmental Adaptation and Conservation Strategies

**DOI:** 10.3390/jof10090632

**Published:** 2024-09-05

**Authors:** Daniel Vasconcelos Rissi, Maham Ijaz, Christiane Baschien

**Affiliations:** Leibniz Institute—DSMZ, German Collection of Microorganisms and Cell Cultures, 38124 Braunschweig, Germany; daniel.rissi@dsmz.de (D.V.R.); maham.ijaz@dsmz.de (M.I.)

**Keywords:** *Nectriaceae* family, fungal lifestyles, global warming, genomic adaptations

## Abstract

This study presents the first genome assembly of the freshwater saprobe fungus *Neonectria lugdunensis* and a comprehensive phylogenomics analysis of the *Nectriaceae* family, examining genomic traits according to fungal lifestyles. The *Nectriaceae* family, one of the largest in Hypocreales, includes fungi with significant ecological roles and economic importance as plant pathogens, endophytes, and saprobes. The phylogenomics analysis identified 2684 single-copy orthologs, providing a robust evolutionary framework for the *Nectriaceae* family. We analyzed the genomic characteristics of 17 *Nectriaceae* genomes, focusing on their carbohydrate-active enzymes (CAZymes), biosynthetic gene clusters (BGCs), and adaptations to environmental temperatures. Our results highlight the adaptation mechanisms of *N. lugdunensis*, emphasizing its capabilities for plant litter degradation and enzyme activity in varying temperatures. The comparative genomics of different *Nectriaceae* lifestyles revealed significant differences in genome size, gene content, repetitive elements, and secondary metabolite production. Endophytes exhibited larger genomes, more effector proteins, and BGCs, while plant pathogens had higher thermo-adapted protein counts, suggesting greater resilience to global warming. In contrast, the freshwater saprobe shows less adaptation to warmer temperatures and is important for conservation goals. This study underscores the importance of understanding fungal genomic adaptations to predict ecosystem impacts and conservation targets in the face of climate change.

## 1. Introduction

The *Nectriaceae* family was introduced in 1865 to accommodate the hypocrealean species, having ascomata that are generally yellow and orange-red to purple and usually change color in potassium hydroxide and lactic acid [1,2]. The family is associated with phialidic asexual morphs that produce conidia, which can range from amerosporous (single-celled) to phragmosporous (multi-celled) [3]. Some genera affiliated with *Nectriaceae* have straight asci and conidia, while those of other genera are strongly curved [1]. The reexamination of *Nectriaceae* fungi has been carried out continuously since 1950 by several taxonomists [4,5,6,7,8,9,10,11,12,13].

The genus *Neonectria* is characterized by well-developed or minute stromata, subglobose to broadly pyriform and gregarious perithecia that are laterally or not collapsing when dry, two- or three-layered perithecial walls, smooth to warted perithecial surfaces, cylindrical to clavate asci with or without an apical ring, and uni- or multiseptated ascospores with spinulose or striate surfaces [14].

*Nectriaceae* is one of the largest families of *Hypocreales* (*Sordariomycetes*, *Ascomycota*) [15], with 167 genera described in Mycobank (9 February 2024, Mycobank). They are frequently found on both living and decaying woody materials, soil, fruiting bodies of other fungi, and insects and occur on various substrates in tropical and subtropical regions around the world [9]. Several of them are documented as endophytes or opportunistic plant pathogens and pathogens of crops and humans [16]. They have the potential to degrade resistant plant material [17] and are used in industrial and commercial applications [3].

The genera *Fusarium* and *Neonectria* are the most studied taxa in *Nectriaceae* due to their relevant economic importance as plant pathogens. The genus *Fusarium* is one of the largest genera in the family *Nectriaceae*, and more than 1000 species have been reported in 2022 [15]. *Fusarium* was included in the top 10 globally most important genera of plant-pathogenic fungi based on its scientific and economic importance [18]. The genus *Neonectria* has 57 species reported in Mycobank (9 February 2024, Mycobank). *Neonectria* species are known for the infection of the branch and trunk, which causes lesions known as cankers; they are normally associated with beech and fruit tree bark cankers [19]. The most well-known *Neonectria* plant pathogens are *N. coccinea* (European beech bark disease); *N. faginata* (American beech bark disease); *N. ditissima* (hardwood canker disease); and *N. punicea* (beech bark disease) [20,21].

*Neonectria* is also a common genus of endophytic fungi [19,22]. Fungal endophytes of plants are widespread and important for host plant health [23]. They spend all or part of their lives residing in healthy plant tissue, often in non-pathogenic mutualistic relationships and often protecting the plant against biotic and abiotic stress and promoting plant growth [22,24,25]. Their presence and abundance are often associated with the developmental stage of the plant, the season, and the environment [25,26]. When in association with the roots, fungal endophytes play important roles in ecosystem processes and nutrient cycling, with beneficial symbiotic relationships with many plants [27,28]. Endophytic fungi can be latent pathogens [29,30], mutualists—for example, mycorrhizal fungi [31]—and/or saprobes [32,33], but should be detected within the tissue of healthy host plants [27,34].

The *Nectriaceae* family also includes fungi with saprobe capabilities, in soil and freshwater [17,35]. Freshwater fungi are believed to have originated from soil fungi approximately 1.5 billion years ago due to the similar microbial machinery found in both groups and evolutionary studies [36,37]. In soil, *Nectriaceae* saprobes have a cosmopolitan distribution around the world and colonize newly dead, organic plant material [38]. Freshwater fungi are characterized as having a partial or complete lifecycle in freshwater environments [39], where they act primarily in the decomposition of leaf litter, particularly aquatic hyphomycetes [40]. Aquatic hyphomycetes are characterized by tetraradiate, branched, and sigmoid conidial shapes that facilitate attachment on the surfaces of aquatic leaf litter [41,42]. However, in aquatic *Nectriaceae*, the typical conidial shape can be clove-shaped with or without minute outgrowths [42,43]. Freshwater ascomycetes possess distinctive adaptations that enable their survival in freshwater environments—for example, their unique lignocellulose enzymes that work by softening leaves and wood, which is particularly fundamental in breaking down wood underwater [36].

According to many studies, in order to adapt to colder environments, certain freshwater fungi have developed unique mechanisms, such as cold-active enzymes and antifreeze proteins [44,45,46,47,48,49]. These characteristics help freshwater fungi to survive in extreme environmental conditions like arctic and subarctic streams and in alpine freshwater streams. However, the escalating temperatures caused by global warming have highlighted the need to comprehend and forecast the reactions of microbial life and ecosystems to severe drought and warm weather occurrences. These events are some of the major environmental stresses experienced by microorganisms such as fungi [50] and can affect the activity of their enzymes and specialized metabolites. For example, the production of specialized metabolites in lichens is affected by light, UV radiation, the altitude, temperature fluctuations, and seasonality [51,52,53]. In addition, global warming may have significant consequences for human health, crop production, and the well-being of forests and other habitats [50]. Therefore, research mechanisms to identify temperature adaptations and the possible impacts of climate change on fungal species have been implemented not only to predict ecosystem impacts but also to target conservation goals [54,55,56].

In this manner, we present the first genome of the saprobe freshwater fungus *Neonectria lugdunensis* and an up-to-date phylogenomics tree of the *Nectriaceae* family, observing the divergences in their genome traits according to the fungal lifestyle. We emphasize their machinery for the breakdown of plant litter and their enzyme capabilities to resist and be active in colder and warmer temperatures, which is an important mechanism as it not only helps to identify their environmental adaptation to temperature fluctuations but may also indicate possible target species for conservation goals in the context of climate change-related impacts.

## 2. Materials and Methods

The *Neonectria lugdunensis* strain was obtained from the internal collection of DSMZ (Leibniz Institute DSMZ, German Collection of Microorganisms and Cell Cultures) with the assigned identifier DSM 113088. Originally, this strain was derived from ascospores of the perithecial morph on a twig of an unknown plant species in Slovakia (GenBank accession number of ITS sequence DQ247777) by Ludmila Marvanová and deposited in the Czech Collection of Microorganisms under CCM F-13783. The fungus was cultivated on agar plates containing 2% malt extract (Feelwell, Gnarrenburg, Germany) using Oxoid brand agar (Basingstoke, UK) at a temperature of 16 °C within a designated cooling facility for DNA extraction. To confirm the taxonomic assignation of the strain cultures, we employed Sanger sequencing of the beta tubulin marker gene. This was accomplished using the pair of primers T1 (5′-AAC-ATG-CGT-GAG-ATT-GTA-AGT-3′) and T2 (5′-TAG-TGA-CCC-TTG-GCC-CAG-TTG-3′). The obtained sequences were manually curated using the Sequencher tool v5.4.6 (http://www.genecodes.com, accessed on 15 November 2023). Subsequently, a BLAST analysis was conducted against the National Center for Biotechnology Information (NCBI) Nucleotide Database (https://www.ncbi.nlm.nih.gov/, accessed on 20 November 2023).

After the taxonomic confirmation of the strain, the biomass was transferred to 1 litter flask each with potato–glucose liquid medium (Carl Roth, Karlsruhe, Germany) and transferred to a 16 °C cooling room over a shaker (Bottmingen, Switzerland) at 120 RPM. The samples were kept in a shaker until sufficient biomass (14 g) for genome extraction was obtained (see Supplementary File). The genomic DNA, with fragment sizes greater than fifteen kilobases, verified with an Agilent Femto Pulse (Agilent, Santa Clara, CA, USA), was subjected to Hifi genome sequencing at Macrogen, the Netherlands. The raw sequence files were obtained from Macrogen using Flye v2.9 [57], and the quality was assessed with Busco v5.2.2 [58] using the ascomycota_odb10 database.

For the comparative genomics of the *Nectriaceae* family, we obtained 17 genomes of *Nectriaceae* from the NCBI database (see Table 1). We aimed to select representative genomes of each genus at random but taking into consideration the lifestyle of each fungal species, and we added all genomes available for the *Neonectria* genus. We also added 2 outgroup genomes for phylogenomics analysis, belonging to the *Ophiocordycipitaceae* family, which is phylogenetically closely related to *Nectriaceae* [36], and the newly assembled genome of *Neonectria lugdunensis*. The genomes were verified according to their genome completeness to ensure values higher than 95%, according to the results obtained with BUSCO and the ascomycota_odb10 database.

An all-vs.-all genome-to-genome alignment and comparison analysis was performed using the DNADIFF program from MUMMER3 [59]. All genomes were aligned and compared against each other to obtain the average nucleotide identity (ANI), and genomes with similarity higher than 95% were considered to be of the same species [60].

The integrative method of RepeatModeler2 [61] was employed to generate a comprehensive *denovo* species-specific repeat library for all genomes. The identification of new repeats by RepeatModeler v2.05 resulted in a library of consensus sequences for each species, combined with repeat annotation carried out using RepeatMasker v4.1.5 [62], via sequence comparison against the Dfam library, generating transposable element results, while soft-masking the genome. The soft-masked genome was used to predict the tRNA sequences determined by tRNAscan-SE 2.0.12 [63] with default parameters for eukaryotic organisms.

The soft-masked genomes from NCBI (17 *Nectriaceae* and 2 outgroup taxa), together with the newly assembled genome of *Neonectria lugdunensis*, were then submitted to the Braker3 v3.0.3 [64] pipeline to perform ab initio gene prediction with the parameters “--esmode” and “--fungus”, using, therefore, the software GeneMarker-ES v4.71_lic [65], to produce de novo hints to train Augustus v3.5.0 [66], which then predicted the amino acid sequences of each genome.

The produced amino acid sequences were used in Orthofinder v3.0.0 [67] with the “-og” option to predict only the single orthologs common to all species in this study, followed by generating sequence clusters. Each cluster was aligned using MAFFT v7.520 [68] with the option“--auto” and trimmed using Trimal v1.4.rev15 [69] with the option “-automated1” to remove poor alignment regions. The phylogenetic clusters were than merged into a unique phylogenetic tree using IQTREE2 v2.2.2.7 [70] with the options –bb 1000 -m TEST. The -m TEST parameter stands for ModelFinder [71], which searches for the best evolutive model for each gene cluster, considering the evolutionary specificity of each gene.

To investigate the genetic potential of the *Nectriaceae* fungi for secondary metabolite production, the AntiSMASH fungal cluster predictor (v7.0.1) [72] was implemented. The presence of genes and clusters associated with the biosynthesis of secondary metabolites was assessed in every genome. Fasta files containing genome sequences were utilized as inputs, with the default search parameter set to “relaxed”. Every additional feature was enabled, including cluster border prediction by utilizing transcription factor binding sites (CASSIS).

The predicted amino acid sequences from the Braker3 pipeline were used for secretome prediction using a combination of tools. To predict where the provided protein sequence possessed a signal peptide, at its N-terminus, which directs the protein towards secretion, we used the consensus sequences obtained from the tools Signalp 6.0 [73] and Targetp2 [74]. In addition, the sequences were also filtered using DeepTMHMM [75] to remove transmembrane domain signals.

The secreted genes were then annotated for carbohydrate-active enzymes (CAZymes) with run_dbCAN4 v4.1.4 (https://github.com/linnabrown/run_dbcan, accessed on 4 November 2023), a standalone tool from the dbCAN3 web server (https://bcb.unl.edu/dbCAN2/, accessed on 4 November 2023), using the tools HMMER v3.3.2 [76], DIAMOND v2.1.8 [77], and dbCAN_sub with the dbCAN3 [78] database v12. The analysis was performed against all 6 available classes of carbohydrate-active enzymes (CAZys): carbohydrate-binding modules (CBMs), glycoside hydrolases (GHs), polysaccharide lyases (PLs), auxiliary activities (AAs), carbohydrate esterases (CEs), and glycosyl transferases (GTs). Only sequences that were identified as CAZy enzymes with 2 or more tools (HMMER, DIAMOND, and dbCAN_sub) were considered for the results, as recommended by dbcan3.

The secreted genes were also annotated for effector genes using EffectorP v3.0 [79], where proteins were predicted to interact or not interact with plants at the apoplast and cytoplasmic level; through the tool, we could observe the secreted effector proteins associated with plant infection by fungi, including the main locus of infection of the protein.

To better understand the adaption of the freshwater hyphomycetes to the environment, proteins with psychrophilic and thermophilic characteristics were predicted using the machine learning tool ThermoProt [80], for the secretome, effector genes, and CAZy genes.

## 3. Results

The newly assembled genome of *Neonectria lugdunensis* has 44.78 Mbp and 97.6% genome completeness with N50 of 44.7 Mbp, showing similar characteristics to many genomes available in NCBI from *Nectriaceae*, as shown in this study (Table 1). We detected 4.38% of repetitive elements in *N. lugdunensis*, where 2.04% were retroelements and 0.33% were DNA transposons. Only 207 sequences of tRNA were identified in *N. lugdunensis*, which is the smallest amount in comparison with the freshwater saprobe fungus *Aquanectria penicillioides* (222) and the soil saprobe *Thelonectria discophora* (225).

*N. lugdunensis* has fewer predicted genes (11,480) than the freshwater saprobe *Aquanectria penicillioides* with 12,575, although this is higher than the soil saprobe *Thelonectria discophora* with 10,364 genes. This result is also reflected in the number of secreted proteins of *N. lugdunensis* being smaller (797) than that of freshwater *A. penicillioides* (820) and higher than that of the soil saprobe *Thelonectria discophora* (655). It is also reflected in the number of effector genes (229 in *N. lugdunensis*, 254 in *A. penicillioides*, and 191 in soil saprobe *T. discophora*) (Table S1). Regarding BGCs, *N. lugdunensis* had higher amounts among saprobe fungi with 48 predicted clusters, followed by the other freshwater fungus *A. penicillioides* with 40 and the soil saprobe *T. discophora* with 33 (Table S2). *N. lugdunensis* was the only freshwater fungus in this study to have a BGC for NRP metallophore.

The species *Ilyonectria robusta* (current name according to Mycobank: *Ramularia robusta*) is known to be a plant pathogen and endophyte. However, the genome strain was isolated as an endophyte (https://mycocosm.jgi.doe.gov/Ilyrob1/Ilyrob1.home.html, accessed on 20 November 2023) and it is therefore presented in this study as such (Table S1).

**Figure 1:**
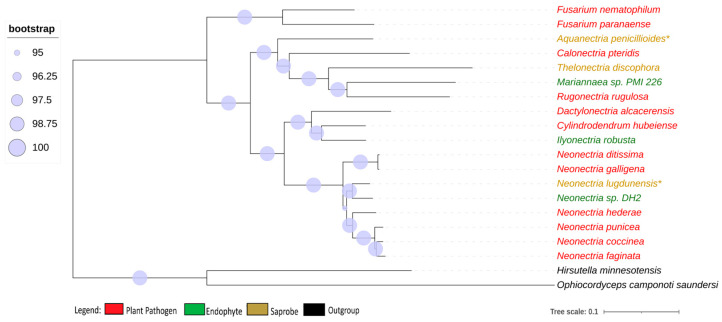
Phylogenomic tree of *Nectriaceae* genomes. Phylogenomic tree of *Nectriaceae* family using 2684 single-copy orthologs genes. The plant pathogen fungus *N. galligena*’s current name is *N. ditissima*. The species in red represent plant pathogens, while those in green are endophytes and those in brown are saprobes. (*) indicates saprobe freshwater fungi. Log-likelihood of consensus tree −18,240,846.991.

We obtained 2684 single-copy ortholog genes, which yielded a robust, well-resolved, and comprehensive phylogeny for the *Nectriaceae* family (Figure 1). The internodes in the tree received a strong bootstrap value higher than 95%, indicating a strongly supported tree that was not particularly associated with the lifestyle of the fungi.

*Neonectria galligena* is nowadays a known synonym for *Neonectria ditissima* and they have been described as being the same species since 1995 [8], although, in the NCBI database, both names are represented with different genomes. The genome-to-genome analysis through ANI confirmed that they are the same species (ANI value of 98.91%), but the enzymatic profile suggests that they are from different strains, presenting different phenotypes, and therefore the synonymy is kept in this study. All other genome comparisons using ANI showed values smaller than 95% between the species’ genomes.

Our results show that the genome length of endophyte organisms was, on average, larger (49.24 Mbp) than that of plant pathogens (48.04 Mbp) and saprobes (46.71 Mbp), despite the largest genome in the study being that of *Dactylonectria alcacerensis*, which is a plant pathogen, with 61.76 Mbp (Figure S1). This value was also reflected in the average number of amino acid sequences predicted, where endophytes (12,971) had the highest, followed by plant pathogens (12,798) and saprobes (11,473) (Figure S2). However, we observed a change in the GC content, where the highest average was found in the plant pathogens compared to saprobe fungi and endophytes (51.54, 51.42, and 51.07, respectively) (Figure S3).

The plant-pathogenic fungi in this study presented more tRNA on average than saprobes and endophytes (218.42, 218.00, and 212.67 respectively).

**Figure 2:**
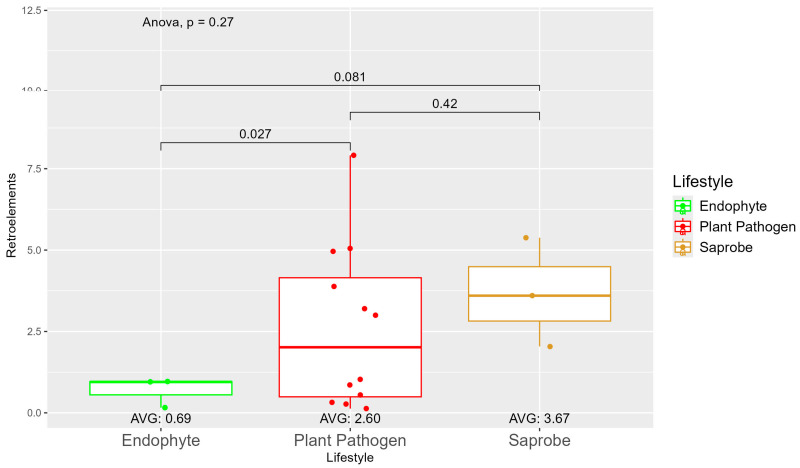
Comparative analysis of retroelement counts among different fungal lifestyles. Distribution of retroelements across three fungal lifestyles: endophytes, plant pathogens, and saprobes. Endophytes: represented in green, the retroelement counts for endophytes are low, with an average count of 0.69. The box plot shows minimal variation, with most values clustering around the average. Plant pathogens: represented in red, plant pathogens exhibit a higher average retroelement count of 2.60. The distribution shows greater variability compared to endophytes, with several outliers, indicating significantly higher counts. Saprobes: represented in brown, they have the highest average retroelement count of 3.67. This group shows larger variability in its retroelement counts.

We observed a large variation in the repetitive elements according to the fungi’ lifestyles (Figure S5), where, on average, the saprobes (7.35) had more repetitive elements than plant pathogens (5.28) and endophytes (3.13). When observing the repetitive element sequences with origins in retroelements, we found statistically significant differences between endophytes (0.69) and plant pathogens (2.60) (Figure 2). The difference in retroelements between saprobes—the largest on average with 3.67—and plant pathogens was not significant; the same was true for saprobes and endophytes. Regarding the repetitive elements classified as DNA transposons (Figure S6), we observed a shift where saprobes had more repetitive DNA transposons on average (0.75) than endophytes (0.68) and plant pathogens (0.53) (Figure S6).

**Figure 3:**
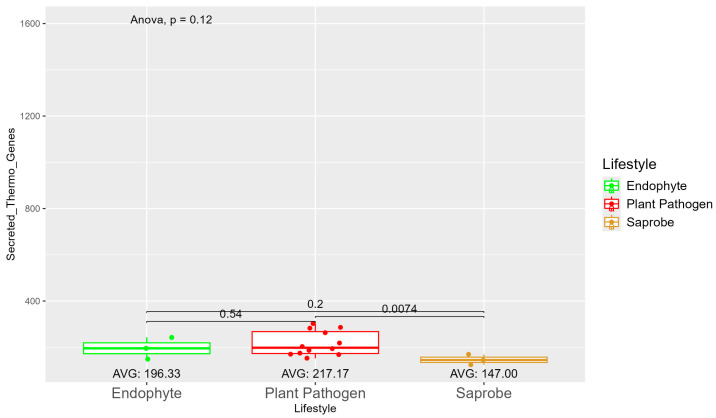
Comparative analysis of secreted thermo gene counts among different fungal lifestyles. Comparative analysis of secreted thermo effector counts among different fungal lifestyles. Endophytes: represented by green, the secreted thermo effector counts for endophytes have an average value of 196.33. The box plot shows moderate variation, with values clustering around the average. Plant pathogens: represented by red, plant pathogens exhibit a higher average secreted thermo effector count of 217.17. The distribution shows greater variability compared to endophytes, with several outliers, indicating significantly higher counts. Saprobes: represented by brown, saprobes have the lowest average secreted thermo effector count of 147.00. This group shows minimal variability in the secreted thermo effector counts. The comparison between plant pathogens and saprobes shows a significant difference (*p* = 0.0074).

The number of genes present in endophytes was also higher than in plant pathogens and saprobes (Table S1). The number of secreted genes was higher in plant pathogens than in endophytes and saprobe fungi (953.33, 907, and 757.33, respectively) (Figure S7). The number of secreted genes with cold properties was higher in endophytes (382.67) than in plant pathogens (359.92) and saprobes (313.33) (Figure S8). On the other hand, the number of secreted genes with thermo-resistance was higher in plant pathogens (217.17) than endophytes (196.33) and the difference was statistically significant among plant pathogens and saprobes (146.00) (Figure 3).

**Figure 4:**
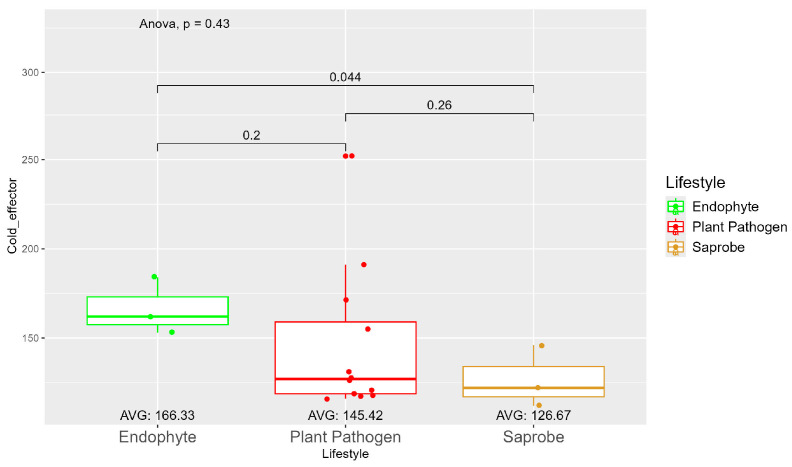
Comparative analysis of secreted cold effector counts among different fungal lifestyles. Differences in effector genes adapted to colder temperatures in endophytes, plant pathogens, and saprobe fungi. Endophytes: represented by green, the cold effector counts for endophytes have an average value of 166.33. The box plot shows moderate variation, with values clustering around the average. Plant pathogens: represented by red, plant pathogens exhibit a lower average cold effector count of 145.42 compared to endophytes. The distribution shows greater variability compared to endophytes, with several outliers, indicating higher counts. Saprobes: represented by brown, saprobes have the lowest average cold effector count of 126.67. This group shows minimal variability in the cold effector counts. Notably, the comparison between plant pathogens and saprobes shows a significant difference (*p* = 0.044).

**Figure 5:**
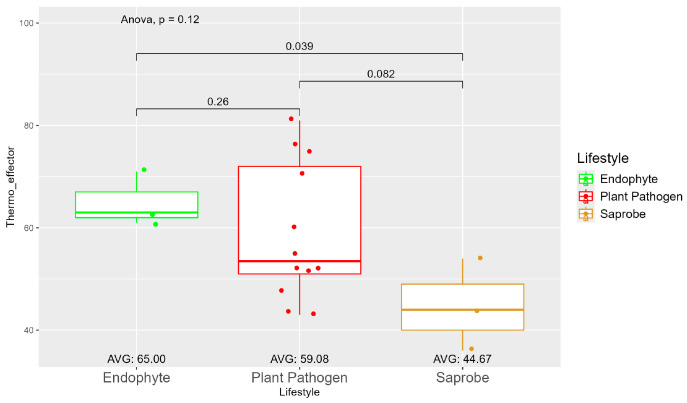
Comparative analysis of thermo effector counts among different fungal lifestyles. Differences in effector genes adapted to higher temperatures in endophytes, plant pathogens, and saprobe fungi. Endophytes: represented by green, the thermo effector counts for endophytes have an average value of 65.00. The box plot shows moderate variation, with values clustering around the average. Plant pathogens: represented by red, plant pathogens exhibit an average thermo effector count of 59.08. The distribution shows greater variability compared to endophytes, with several outliers indicating higher counts. Saprobes: represented by brown, saprobes have the lowest average thermo effector count of 44.67. This group shows minimal variability in its thermo effector counts. Notably, the comparison between endophytes and plant pathogens shows a significant difference (*p* = 0.039).

The average number of effector proteins secreted by endophytes (278.33) is higher than in plant pathogens (268.17) and saprobes (224.67) (Figure S9). The temperature adaptation for effector genes shows statistical significance between endophytes and saprobes for cold and thermal adaptation, where the number of effector genes adapted to cold was higher in endophytes (166.33), followed by plant pathogens (145.42) and saprobes (126.67) (Figure 4). The endophytes also showed higher potential in adapting to warmer temperatures than plant pathogens and saprobes (65.00, 59.08, and 44.67, respectively) (Figure 5).

Endophytes have been shown to have more effector genes on average with apoplastic characteristics (237.00) than plant pathogens (223.92) and saprobes (190.67) (Figure S10). On the other hand, the difference in the number of effector genes with cytoplasmatic characteristics is shown to be statistically significant between endophytes (99.93 on average) and saprobes (71.00 on average); despite this, plant pathogens (101.58) had higher numbers than endophytes and saprobes (Figure S11).

The average number of CAZy genes is the highest in plant pathogens (286.75), followed by endophytes (269.33) and saprobes (232.33) (Figure S12). These findings suggest that while there is some variation in the CAZy gene counts among these fungal lifestyles, these differences are not statistically significant, indicating a relatively similar distribution of CAZy genes across endophytes, plant pathogens, and saprobes. Regarding temperature adaptation, the average number of secreted thermo-adapted CAZy genes is higher in plant pathogens (41.83) compared to endophytes (40.00) and saprobes (28.67) (Figure S13). These results suggest that while there are some variations in the thermo-adapted CAZy gene counts, these differences are not statistically significant across the three fungal lifestyles analyzed. The average number of cold-adapted CAZy genes is the highest in plant pathogens (129.58), followed by endophytes (123.33) and saprobes (113.67) (Figure S14). These results suggest that the number of cold-adapted CAZy genes is relatively similar across these fungal lifestyles, with no statistically significant variation observed.

Regarding biosynthetic gene clusters, endophytes were shown to produce, on average, more biosynthetic metabolites based on the cluster results than plant pathogens and saprobes (54, 53.25, and 40.33, respectively) (Figure S15).

**Figure 6:**
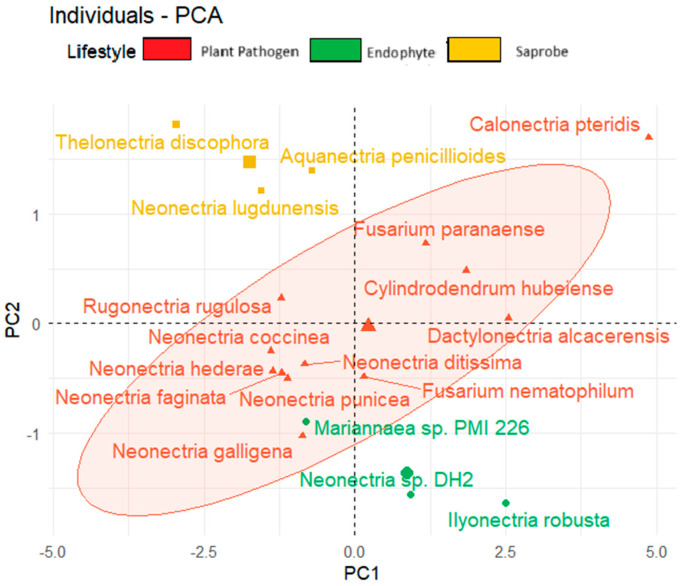
Principal component analysis (PCA) of fungal species based on lifestyle considering secreted genes, effector proteins, and total biosynthetic gene clusters.

We observed also the tendency to form clusters based on the fungal lifestyle when considering the temperature adaptation of the CAZy and effector genes and the total BGCs (Figure 6). In the upper corner region of the figure, we show saprobe organisms, while endophytes are present in the lower region of the graph. The plant pathogens tend to be positioned in the central area of the graph.

## 4. Discussion

Endophytes exist within plant tissue without causing apparent harm to the host, whereas plant pathogens infect and cause illness in plants. Both of these groups work closely with living plant cells, overcoming the plant’s immunological defenses and adapting to its metabolic environment [80,81,82,83]. These interactions need a complex set of genes that create enzymes, toxins, and other compounds that aid the invasion of plant cells, suppressing the plant immune system’s responses, and manipulating the plant’s metabolism. As a result, their genomes frequently contain genes for complicated secondary metabolite production, a variety of transporters, and genes that confer resistance to plant defense mechanisms [84,85]. On the other hand, saprobe fungi, as decomposers of organic material, require a different set of enzymatic tools focused primarily on breaking down cellulose, lignin, and other plant structural components. While this still requires a diverse set of enzymes, the interaction with non-living material is less complex than manipulating and responding to living cells; therefore, it is expected that saprobes have fewer genes than endophytes and plant pathogens [86,87,88].

The repetitive elements are microsatellite regions or, more often, transposable elements (transposons), which increase their number in the genome, mainly due to cellular stress, which is not a characteristic of endophyte environments [89,90,91]. This corroborates our findings of fewer repetitive elements in endophyte genomes, which is likely influenced by their lifestyle, since being endophytic is a very cost-effective strategy. When establishing themselves within plants, they are shielded from abiotic and biotic challenges, being less subjected to stress, which is a factor involved in the multiplication of transposable elements [92].

Our study confirms the results of Queiroz and Santana, 2020 [93], where the number of repetitive elements was a key factor in distinguishing between pathogenic and endophytic fungi, despite its lack of statistical significance. Several studies have found transposable elements to be abundant in plant-pathogenic fungi [94,95,96]. In our study, this case was even better observed with retrotransposons, where the difference in occurrence between endophytes and plant pathogens was statistically significant. The amount of retroelements identified in endophytes is smaller than in saprobe and statistically significantly smaller than in plant pathogens.

Transposable elements contribute significantly to genomic plasticity, which is crucial in adapting to host defenses. For instance, in the case of the plant pathogen *Verticillium dahliae*, TEs exert an influence on gene expression variations that are essential for pathogenicity, enabling the fungus to adapt rapidly to host-induced stresses or resistance [97]. Meanwhile, saprobe fungi are not known for presenting large amounts of TEs [95,98,99], since they have a lesser requirement for adaptation to host living cells and may reside in less stressful environments [100,101,102], although the higher number of TEs found for saprobes in our study could indicate potential adaptation to more dynamic or challenging environmental conditions [95].

The secretome of fungi, which includes CAZy enzymes, plays a pivotal role in the interaction between fungi and plants. These enzymes are crucial not only in breaking down the complex polysaccharides found in plant cell walls, which aids in nutrient acquisition and infection processes, but also in helping fungi to evade plant immune responses, facilitating their survival and propagation in various environments [103,104,105]. The CAZymes of classes CE, GH, and PL are often referred to as plant-litter-degrading enzymes because they play crucial roles in the degradation of plant biomass by fungi and bacteria [106].

In our study, saprobe fungi produced fewer secreted CAZy enzymes than endophytes and plant pathogens. According to Zhao et al. [107], saprobe fungi are expected to only degrade plant litter complex polysaccharides from dead material, without having to interact with the plant’s living cell defenses, and, due to this, they typically require a narrower range of enzymes. In contrast, endophytes and pathogens interact directly with living plant tissue, requiring a broader array of enzymes not only to break down living plant tissue but also evade or suppress plant immune responses and successfully infect their hosts. These interactions demand more specialized enzymatic functions, such as those that degrade pectin, hemicellulose, and other plant cell wall components under varying physiological conditions, so as to successfully establish mutualistic relationships with their hosts [88,108,109,110].

CAZy enzymes, when secreted, perform several functions beyond the degradation of plant cell walls. They also play roles in promoting attachment, invasion, colonization, and nutrient acquisition from hosts, which is why numerous studies suggest that endophytes and plant pathogens can produce the same leaf-degrading enzymes as closely related saprobic fungi [88,106,109,111,112,113,114,115,116].

Pathogens and endophytes must overcome various layers of plant protection to successfully infect the plant [117,118]. The first immunological response of the plant is through receptor-like kinases that can detect pathogen-associated molecular patterns called microbe-associated molecular patterns (MAMPs), which activate the MAMP-triggered immunity or pathogen-triggered immunity, which is effective against a wide range of microorganisms [119,120,121,122]. However, microorganisms can bypass this mechanism by producing effector proteins that alter the cellular processes in the host, leading to effector-triggered susceptibility [120,123,124,125,126]. In addition, the plant has a second layer of receptors called resistant proteins or R proteins, which also recognize patterns in effector proteins in the host, generating a resistance response that promotes the death of infected cells containing the infection [120,127,128,129,130,131]. Hence, effector proteins have distinct properties enabling them to act in the host extracellular space (apoplast) and intracellular space (cytoplasm) during infection. Cytoplasmic effectors have a larger proportion of positively charged amino acids, whereas apoplastic effectors are enriched in cysteine residues [79]. Therefore, fungi with frequent interactions with the plant are expected to possess a large number of effector genes to effectively counteract the plant’s immune system.

However, the cell defense system is not the only obstacle to the survival and adaptation of fungi. The environmental conditions play a major role in protein synthesis, sporulation, and the diversity of their communities. Currently, the world is undergoing significant and rapid climate change caused by human activities, also referred to as global warming. This has resulted in a rise in temperatures, which are presently approximately 1.5 °C higher than they were during the pre-industrial period [132]. This even raises concerns about the adaptation of fungal species with pathogenic capabilities, such as those that infect plants, since an increase in plant diseases in crops is expected under projected climate change scenarios [132,133,134].

In our study, the pathogenic fungi from the *Nectriaceae* family had more secreted enzymes adapted to higher temperatures than endophytes and saprobes, which indicates their higher potential to adapt to global warming situations. The potential for growth and infection is observed in many of the most economically important plant pathogens from the *Nectriaceae* family, belonging to the genera *Neonectria* and *Fusarium* [135,136,137,138,139,140,141]. In our study, plant pathogens presented less cold-adapted and more thermo-adapted proteins, indicating the potential to perform better in higher temperatures, which reinforces the findings of [137], showing that phytopathogens from the *Nectriaceae* order are organisms that may prevail in global warming, increasing their distribution and impact.

On the other hand, in some cases, the temperature can be a comprehensive factor that impacts cellular metabolism and functions. This encompasses essential biochemical parameters, such as the rates at which reactions occur, molecule binding, and the flexibility of cell membranes [132,142]. As an example, freshwater saprobe fungal communities are susceptible to the effects of climate change in terms of their species composition and abundance; this, therefore, impacts freshwater ecosystems’ functions [143,144,145,146]. They are richer in cold regions or environments and have genome machinery that is more adapted to colder temperatures [54,147]. Therefore, the increase in the temperature due to global warming could cause a reduction in their activity and could negatively impact energy, carbon, and nutrient cycling, threatening the delivery of ecosystem services to higher organisms [148,149,150,151].

The newly assembled genome of *Neonectria lugdunensis*, presented in this study, was first isolated from a submerged decaying twig in a stream bed (personal communication, L. Marvanová) and is commonly reported in aquatic ecology studies [152] but has also been identified in soil [153]. *N. lugdunensis* has been shown to be resistant to long droughts at 25 °C and has the potential to endure even higher temperatures for a limited time, as shown in [151]. Other freshwater fungi have better adaptation to colder temperatures, showing growth peaks at temperatures between 15 °C and 25 °C [144,151].

The small amount of thermo-adapted proteins observed in the saprobe fungi in this study is concerning as the increase in the worldwide temperature could also lead to a decline in saprobe biodiversity, growth, and survival. The temperature is one of the most important factors influencing the structure of saprobe communities, and this may have severe consequences in the ecological process of decomposition [154]. Therefore, the monitoring of their biodiversity and the implementation of conservation efforts are vital to preserve the biodiversity of saprobe fungi.

Protein synthesis is a central cellular process that is partly regulated by the availability of tRNA molecules. tRNAs can pre-present in the genome and spread in multiple families in multiple copies or single genes [155]. They are vital for the breakdown of organic matter and the synthesis of proteins and metabolites that can interact with living organisms. In endophytes and plant pathogens, they are essential for the organism’s virulence and the evasion of host defense and are involved in pathogenicity [156,157,158]. Therefore, the number of copies of tRNA and their variability may be utilized to predict the efficiency with which genes may be translated. This allows for the estimation of protein synthesis rates, cells’ responsiveness to external influences, and, eventually, evolutionary adaptations to novel environments, and it has been considered a determinant of lifestyle transitions among basidiomycetes [155,159]. In our data, tRNA was found to be significant in different fungal lifestyles. The plant-pathogenic fungi in this study presented more tRNAs on average than saprobes and endophytes, in contrast to what was found in other studies [80,81].

Fungi have developed distinct mechanisms for the production of CAZymes and secondary metabolites to suit their lifestyles [160,161,162,163]. Secondary metabolites are primarily encoded by biosynthetic gene clusters (BGCs), which are jointly controlled and located very close together in a certain genome region; as a result, the BGCs responsible for a given specialized metabolite are either “silent” or upregulated [51,164,165,166].

These metabolites play a crucial role in several key adaptive processes associated with ecological interactions and stress responses within their environment and are not directly associated with the growth or reproduction of fungi [164,167,168,169], being often linked to communication and the defense of and/or attacks against their surrounding organisms or hosts [51]. Therefore, they are expected to be present in higher quantities in endophytes and phytopathogens, as presented in this study.

Many secondary metabolites act as pathogenicity factors and have detrimental impacts on host health [163], largely due to the production of mycotoxins—toxic secondary metabolites encoded by BGCs. Mycotoxins can act as virulence factors, weakening or killing host plants and aiding colonization [170,171,172]. The *Nectriaceae* fungi have been noted for their biological activity; for their parasitism on plants, fungi, and insects; and as producers of antibiotics and/or mycotoxins [173]. However, not much research has been conducted in the study of the mycotoxins of the genus *Neonectria* during plant infection.

Non-ribosomal peptide synthetases (NRPS) and polyketide synthases (PKS) are two types of large, modular enzyme complexes (megasynthases) involved in the biosynthesis of non-ribosomal peptides and polyketides, respectively. NRPS create molecules by joining amino acids. In contrast, PKS create molecules by joining acyl groups to form large polypeptide chains, which are responsible for many different catalytic domains, each performing a different chemical reaction. This allows them to create highly complex and diverse molecules [51]. In plant-associated fungi, NRPS and PKS have distinct functions in the synthesis of phytotoxins, mycotoxins, and antibiotics [174]. According to Yoder (2001) [175], numerous virulence factors have been identified in NRPS clusters, demonstrating them as necessary mechanisms in fungal pathogenesis. They were found in higher concentrations in plant pathogens and endophytes in this study and in lower amounts in freshwater saprobes.

Terpene cyclases are enzymes found in plants and microorganisms that help to form monoterpenes, sesquiterpenes, and diterpenes by converting prenyl diphosphate chains. Terpenoids come from isopentenyl diphosphate (IPP), which prenyl transferases modify to create geranyl diphosphate (GPP), farnesyl diphosphate (FPP), and geranyl diphosphate (GGPP). These compounds serve as starting points for the production of monoterpenes (from GPP), sesquiterpenes (from FPP), and diterpenes (from GGPP) with the help of terpene cyclase enzymes [176,177,178]. Currently, over 80,000 terpenoids are known [179]. Our study found the highest number of terpene clusters in plant-pathogenic fungi. The terpene is known in pathogenic fungi for producing many mycotoxins of the class sesquiterpenoids, which play a vital role in fungal virulence [180]. Terpenes can also have effects on fungal growth and protective effects against oxidative stress and UV radiation via the production of carotenoids [178,181,182]. Different environmental factors, including light and temperature, have been shown to change the production levels and compositions of carotenoids [183].

Ribosomally synthesized and post-translationally modified peptides (RiPPs) were found without many variations in this study. Fungal RiPPs are an increasingly important group of natural products, known for their powerful biological activity and antimicrobial, antifungal, or antiviral properties, with many scientific applications [184]. They are produced through a straightforward process. The initial step involves the synthesis of a precursor peptide by the ribosome, which consists of leader, core, and follower amino acid sequences. Subsequently, the core sequence undergoes specific post-translational modifications, guided by the leader and follower sequences. The final bioactive RiPP is released after these sequences are removed [185].

Metal ions play a vital role in numerous enzymatic processes, but their excess can be detrimental to the growth and development of various organisms [186]. Research has shown that siderophores, originally known for their iron-binding capacity, can bind to a variety of metals. This multi-faceted functionality has prompted the broader classification of these compounds as “metallophores”, referring to secondary metabolites capable of binding a range of metal (loid) cations [187,188]. Metallophores are low-molecular-weight organic ligands that facilitate the delivery of essential metal ions to an organism, while the organism regulates the production and release of these ligands based on its metal ion requirements [187]. In our study, we detected siderophores binding with transporter nickel (NI–siderophore) in only three species (*C. pteridis*, *I. robusta*, and *F. nematophilium*), while metallophores (NRP metallophores) were found in 11 species in this study, with one cluster per organism.

The BGC indole is a volatile compound that has been associated with effective fungicides to target continuous fungal infections [189,190]. It was not identified in the saprobe genomes in this study, showing that it might be a crucial cluster for fungal infection and virulence in plants.

The phosphate compounds produced in BGCs are chemicals with phosphorus–carbon bonds with the general chemical formula C−PO(OH)2 or C−PO(OR)2, where R is an alkyl or aryl functional group [191]. They have a wide range of bioactivity, such as antibiotic, antiviral, pesticide, and antiparasitic effects, and approximately 15% of all phosphonate natural products are commercialized [192]. In our study, they were found in five plant pathogens and one endophyte, showing them to be an additional feature in fungal infections.

Isocyanides, also known as isonitriles, are a group of microbial secondary metabolites that have been extensively studied due to their wide range of pharmacological applications, such as antifungal, antibacterial, antitumor, and antiprotozoal applications [193,194,195,196]. In addition, they play an important role in the pathogenesis of insect, plant, and human diseases [196,197,198], showing them to be an additional tool for fungal infection, found in 10 plant pathogen fungi and two endophytes in this study. These compounds are distinguished by the highly reactive isocyanide functional group (R ≡ N+ − C−), which originates from the conversion of specific amino acids within the compound [199].

For endophytes, mycotoxins can also cause herbivory limitations. Ergot alkaloids produced by endophytes limit herbivory, protecting the plant and increasing the fitness of both the plant and the fungus [200]. In addition, endophyte mycotoxins can also prevent their predation by insects, aiding in competition against these pests [201] and, as a consequence, protecting the plant and the fungus itself from predation. Biosynthetic gene clusters such as betalactone are known to be antiviral heterocyclic compounds contributing to biocontrol activity and, in addition, acting in insect immune suppression [200,201,202]. This might be important for endophytes and plant pathogens to avoid the predation of the leaf and competition by other organisms.

## 5. Conclusions

We found that endophytes and plant pathogen fungi carry more effector genes and genes dedicated to secondary metabolism than saprobes, likely due to their stronger interactions with the defense mechanisms of living cells in plants.

Despite not finding many variables with statistical significance besides the numbers of retroelements and thermo-effector genes, we can still differentiate among different fungal lifestyles based on the combination of characteristics associated with plant interactions with enzymes and metabolites shown in this study. This suggests that we can determine the lifestyles of fungi based on these data, and, therefore, machine learning algorithms could be applied for efficient lifestyle prediction in larger datasets. In addition, the low abundance of effector genes adapted to higher temperatures found in the freshwater saprobes in this study may suggest that they are important organisms in targeting preservation goals due to global warming. In contrast, the higher number of thermo-adapted effector genes in plant pathogens suggests a greater capacity to enhance their bioactivity as temperatures rise and therefore the potential to escalate the economic impacts of these fungi when proliferating in agricultural systems.

Many aspects still need to be explored regarding *Nectriaceae* within the genome, the laboratory, and the field, such as the secondary metabolites during the fungal infection of plants and their adaptation to different temperatures due to global warming. In the next few years, many plant pathologists may study effector proteins and metabolites that contribute to effector functions in plant cell walls to better understand the mechanisms of infection of fungi and reduce their economic impacts. In the same direction, freshwater fungi show great potential to be explored in enzymes adapted to cold temperatures and secondary metabolites, with biotechnological importance in different areas.

## Figures and Tables

**Table 1:** Basal genome information of the *Nectriaceae* family in this study. The plant pathogen fungus *N. galligena*’s current name is *N. ditissima*.

Species	Genome Length (Mbp)	Genome Compl. (%)	GC Content (%)	N50	Assembly Accession
*Aquanectria penicillioides*	53.76	95.0%	47.94	4.93 Mbp	GCA_003415625.1
*Calonectria pteridis*	58.37	97.7%	50.21	3.2 Mb	GCA_022837005.1
*Cylindrodendrum hubeiense*	48.81	96.5%	51.81	85.2 Kb	GCA_014621425.1
*Dactylonectria alcacerensis*	61.76	97.9%	49.86	4.3 Mb	GCA_029931735.1
*Fusarium nematophilum*	50.82	96.4%	53.92	148.30 Kb	GCA_033030565.1
*Fusarium paranaense*	53.40	98.2%	49.21	859.2 Kb	GCA_027886155.1
*Ilyonectria robusta*	59.64	97.5%	51.68	1.2 Mb	GCF_021365365.1
*Mariannaea* sp. PMI 226	42.25	97.7%	48.55	2.9 Mb	GCA_024336345.1
*Neonectria coccinea*	42.74	97.3%	51.65	178.8 Kb	GCA_019137265.1
*Neonectria ditissima*	44.95	97.5%	51.83	1.8 Mb	GCA_001305505.1
*Neonectria faginata*	42.94	97.4%	52.48	4.4 Mb	GCA_030864175.1
*Neonectria galligena*	41.00	96.7%	53.9	31.3 Kb	GCA_013759035.1
*Neonectria hederae*	43.28	97.5%	49.43	248.9 Kb	GCA_003385265.1
*Neonectria lugdunensis*	44.78	97.6%	52.17	44.7 Mb	GCA_041721585.1
*Neonectria punicea*	41.47	96.8%	52.72	41.4 Mb	GCA_003385315.1
*Neonectria* sp. DH2	45.82	94.8%	52.99	45.8 Mb	GCA_003934905.1
*Rugonectria rugulosa*	46.95	97.6%	51.43	56.0 Kb	GCA_023509875.1
*Thelonectria discophora*	41.60	97.8%	54.16	41.6 Mb	GCA_911649645.1

## Data Availability

Data are contained within the article and Supplementary Materials. Tables S1 and S2 with the number of features annotated are available at https://zenodo.org/doi/10.5281/zenodo.13333785 (accessed on 16 August 2024), and the genome sequences used in this research have been deposited in NCBI’s GenBank (their accession numbers can be found in Table 1).

## Supplementary Materials

The following supporting information can be downloaded at: https://www.mdpi.com/article/10.3390/jof10090632/s1, Figure S1: Comparative analysis of genome length among different fungal lifestyles; Figure S2: Comparative analysis of amino acid sequence counts among different fungal lifestyles; Figure S3: comparative analysis of GC content among different fungal lifestyles; Figure S4: Comparative analysis of tRNA counts among different fungal lifestyles; Figure S5: Comparative analysis of repetitive element counts among different fungal lifestyles; Figure S6: Comparative analysis of DNA transposon counts among different fungal lifestyles; Figure S7: Comparative analysis of secreted gene counts among different fungal lifestyles; Figure S8: Comparative Analysis of Secreted Cold gene Counts Among Different Fungal Lifestyles; Figure S9: Comparative analysis of effector gene counts among different fungal lifestyles; Figure S10: Comparative analysis of apoplastic protein counts among different fungal lifestyles; Figure S11: Comparative analysis of cytoplasmic protein counts among different fungal lifestyles; Figure S12: Comparative analysis of CAZy gene counts among different fungal lifestyles; Figure S13: Comparative analysis of thermo-adapted CAZy gene counts among different fungal lifestyles; Figure S14: Comparative analysis of cold-adapted CAZy gene counts among different fungal lifestyles; Figure S15: Comparative analysis of total BGC counts among different fungal lifestyles; Table S1: Genome annotation for every species in this study; Table S2: Genome annotation for biosynthetic gene clusters identified in the genomes.
