# Supplementary material for: Comparative Genomics of Fungi in Nectriaceae Reveals Their Environmental Adaptation and Conservation Strategies
Source: J Fungi (Basel). 2024 Sep 5;10(9):632. doi: 10.3390/jof10090632 (PMC11433043; doi:10.3390/jof10090632)
Supplement: Supplementary file 1 [file jof-10-00632-s001.zip › Figures S1–S15.pdf]

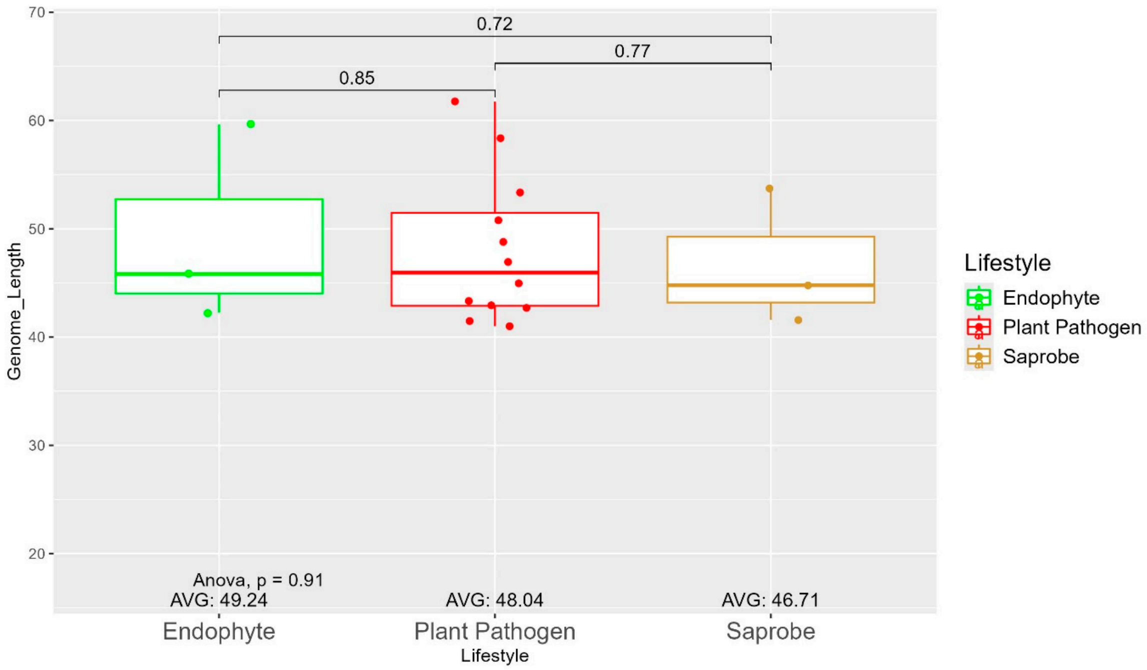

Figure S1: Comparative analysis of genome length among different fungal lifestyles.
Genome lengths across three fungal lifestyles: Endophyte, Plant Pathogen, and Saprobes. **Endophyte**: Represented by green, the genome lengths for endophytes have an average value of 49.24. The box plot shows moderate variation with values clustering around the average. **Plant Pathogen**: Represented by red, plant pathogens exhibit an average genome length of 48.04. The distribution shows greater variability compared to endophytes, with several outliers indicating higher values. **Saprobe**: Represented by brown, saprobes have an average genome length of 46.71. This group shows minimal variability in genome length.

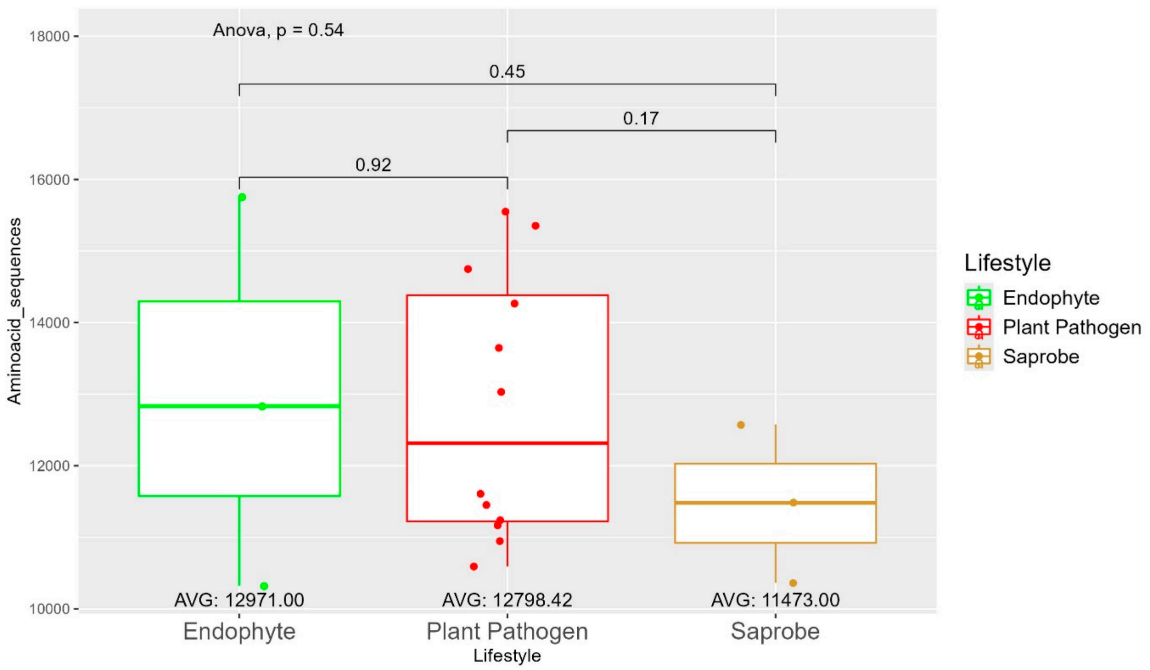

Figure S2: Comparative analysis of amino acid sequence counts among different fungal lifestyles
The distribution of amino acid sequence counts across three fungal lifestyles: Endophyte, Plant Pathogen, and Saprobes. **Endophyte**: Represented by green, the amino acid sequence counts for endophytes have an average value of 12,971.00. The box plot shows moderate variation with values clustering around the average. **Plant Pathogen**: Represented by red, plant pathogens exhibit an average amino acid sequence count of 12,798.42. The distribution shows greater variability compared to endophytes, with several outliers indicating higher values. **Saprobe**: Represented by brown, saprobes have an average amino acid sequence count of 11,473.00. This group shows minimal variability in amino acid sequence counts.

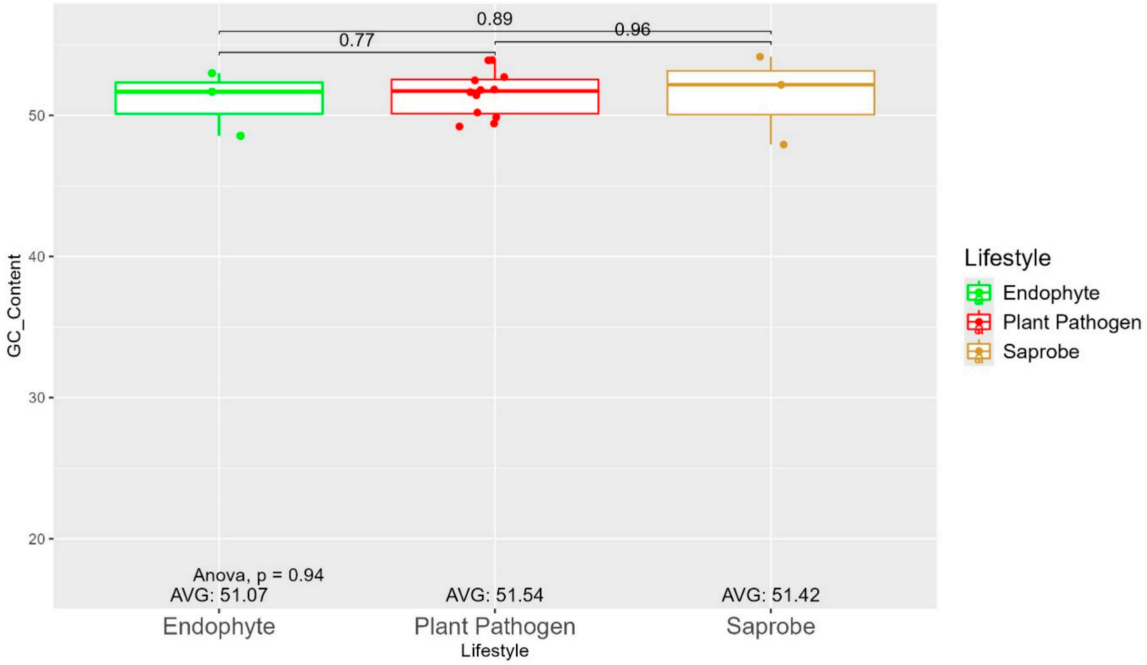

Figure S3: comparative analysis of GC content among different fungal lifestyles

The distribution of GC content across three fungal lifestyles: Endophyte, Plant Pathogen, and Saprobes. **Endophyte**: Represented by green, the GC content for endophytes has an average value of 51.07. The box plot shows moderate variation with values clustering around the average. **Plant Pathogen**: Represented by red, plant pathogens exhibit an average GC content of 51.54. The distribution shows minimal variability compared to endophytes. **Saprobe**: Represented by brown, saprobes have an average GC content of 51.42. This group shows minimal variability in GC content.

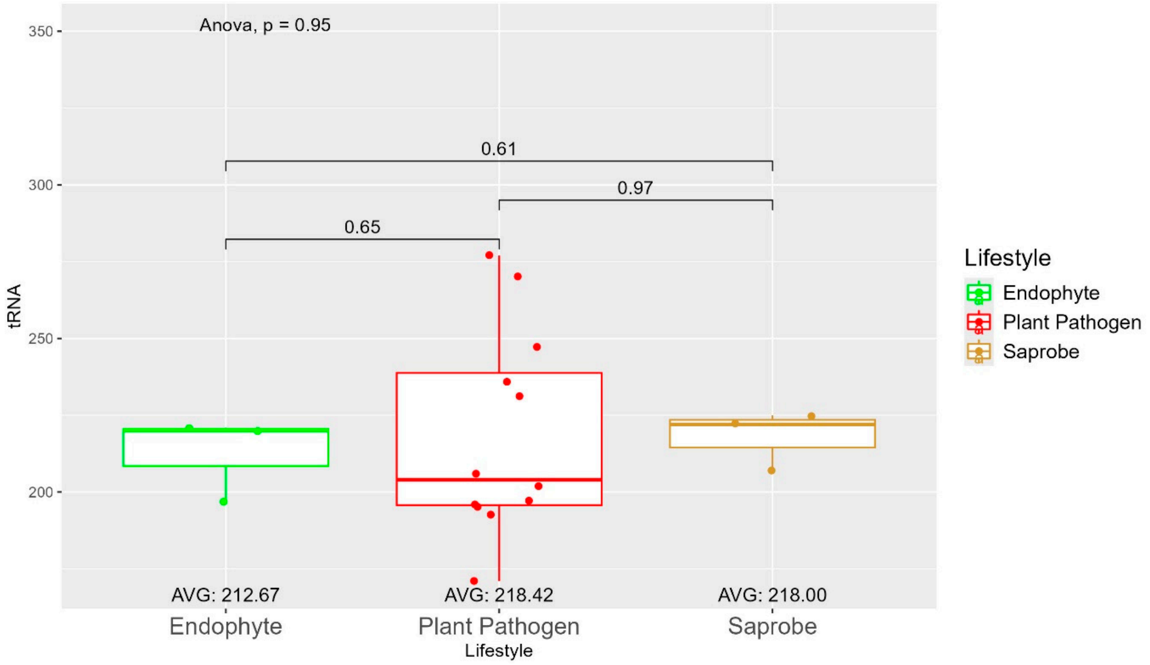

Figure S4: Comparative analysis of tRNA counts among different fungal lifestyles
The distribution of tRNA counts across three fungal lifestyles: Endophyte, Plant Pathogen, and Saprobes. **Endophyte**: Represented by green, the tRNA counts for endophytes have an average value of 212.67. The box plot shows moderate variation with values clustering around the average. **Plant Pathogen**: Represented by red, plant pathogens exhibit an average tRNA count of 218.42. The distribution shows greater variability compared to endophytes, with several outliers indicating higher values. **Saprobe**: Represented by brown, saprobes have an average tRNA count of 218.00. This group shows minimal variability in tRNA counts.

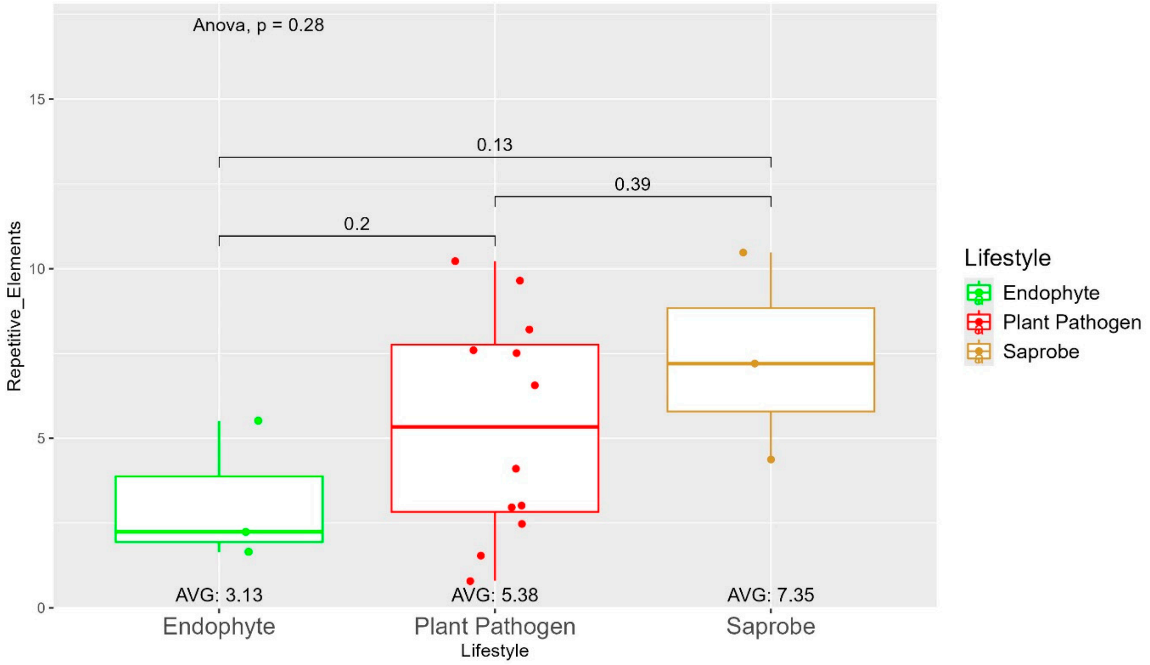

Figure S5: Comparative analysis of repetitive element counts among different fungal lifestyles
The distribution of repetitive element counts across three fungal lifestyles: Endophyte, Plant Pathogen, and Saprobes. **Endophyte**: Represented by green, the repetitive element counts for endophytes have an average value of 3.13. The box plot shows moderate variation with values clustering around the average. **Plant Pathogen**: Represented by red, plant pathogens exhibit an average repetitive element count of 5.38. The distribution shows greater variability compared to endophytes, with several outliers indicating higher values. **Saprobe**: Represented by brown, saprobes have the highest average repetitive element count of 7.35. This group shows moderate variability in repetitive element counts.

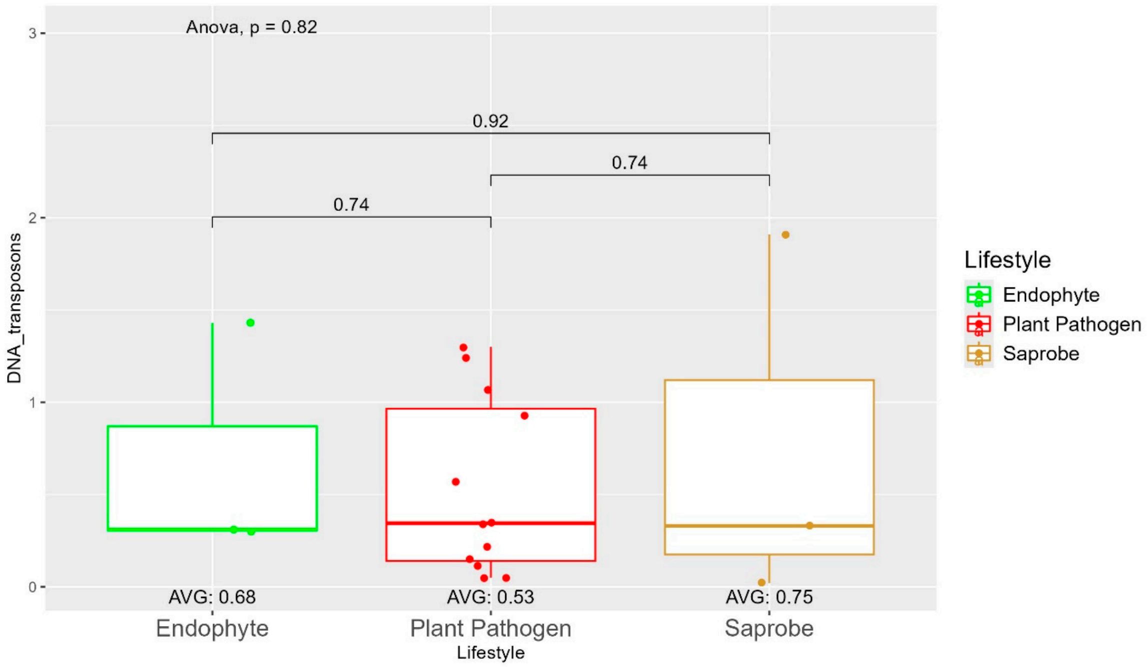

Figure S6: Comparative analysis of DNA transposon counts among different fungal lifestyles

The distribution of DNA transposon counts across three fungal lifestyles: Endophyte, Plant Pathogen, and Saprobes. **Endophyte**: Represented by green, the DNA transposon counts for endophytes have an average value of 0.68. The box plot shows moderate variation with values clustering around the average. **Plant Pathogen**: Represented by red, plant pathogens exhibit an average DNA transposon count of 0.53. The distribution shows moderate variability compared to endophytes. **Saprobe**: Represented by brown, saprobes have the highest average DNA transposon count of 0.75. This group shows greater variability in DNA transposon counts.

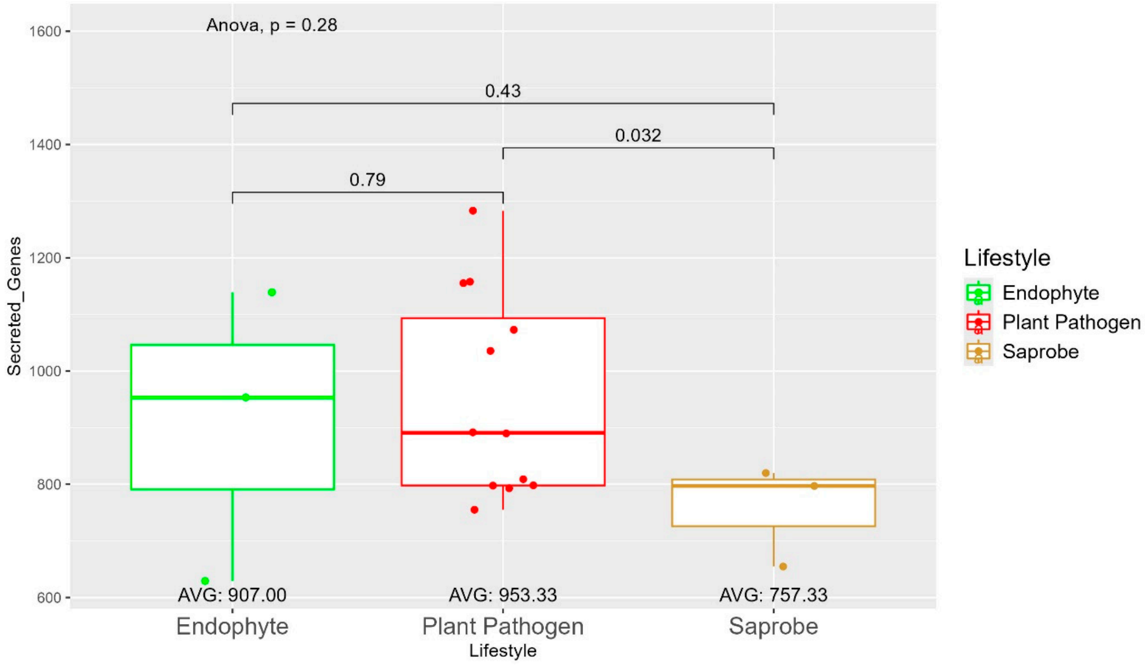

Figure S7: Comparative analysis of secreted gene counts among different fungal lifestyles

The distribution of secreted CAZy (Carbohydrate-Active enZYmes) counts across three fungal lifestyles: Endophyte, Plant Pathogen, and Saprobes. **Endophyte**: Represented by green, the secreted CAZy counts for endophytes have an average value of 907.00. The box plot shows moderate variation with values clustering around the average. **Plant Pathogen**: Represented by red, plant pathogens exhibit an average secreted CAZy count of 953.33. The distribution shows greater variability compared to endophytes, with several outliers indicating higher values. **Saprobe**: Represented by brown, saprobes have an average secreted CAZy count of 757.33. This group shows moderate variability in secreted CAZy counts.

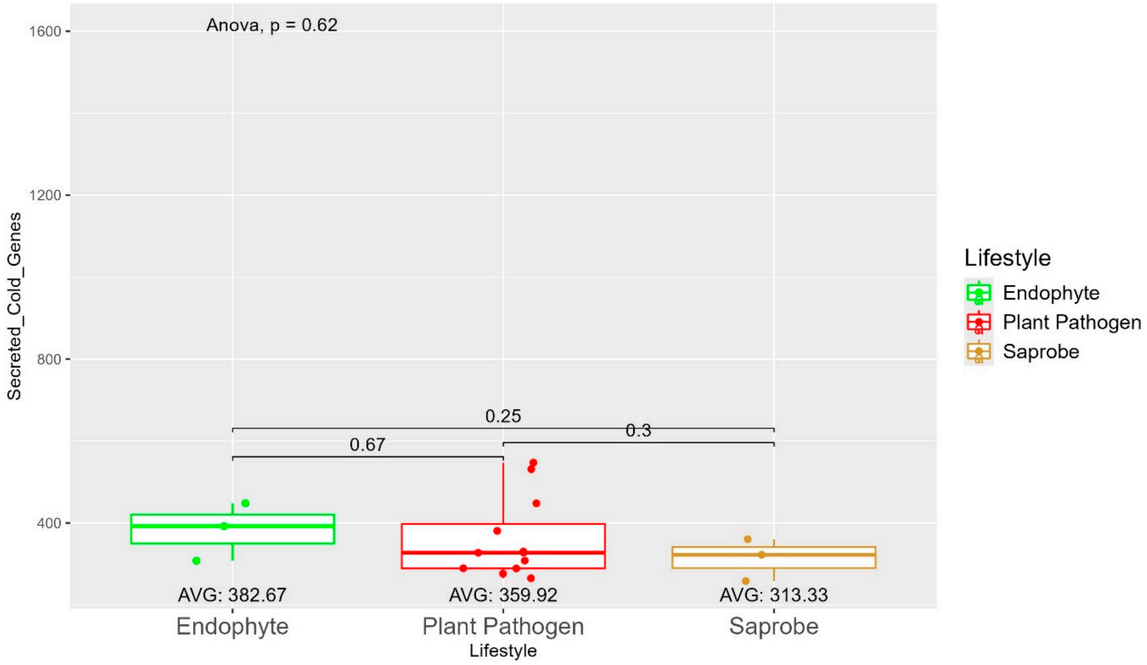

Figure S8: Comparative Analysis of Secreted Cold gene Counts Among Different Fungal Lifestyles
The distribution of secreted cold effector counts across three fungal lifestyles: Endophyte, Plant Pathogen, and Saprobes. **Endophyte**: Represented by green, the secreted cold effector counts for endophytes have an average value of 382.67. The box plot shows moderate variation with values clustering around the average. **Plant Pathogen**: Represented by red, plant pathogens exhibit an average secreted cold effector count of 359.92. The distribution shows greater variability compared to endophytes, with several outliers indicating higher values. **Saprobe**: Represented by brown, saprobes have the lowest average secreted cold effector count of 313.33. This group shows minimal variability in secreted cold effector counts.

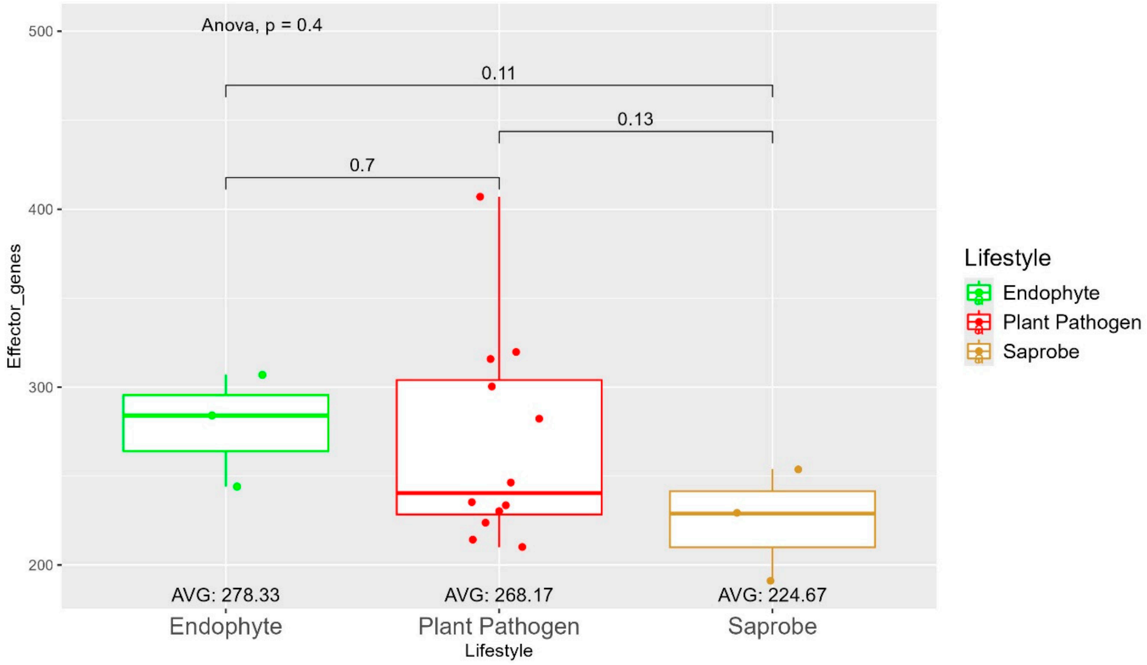

Figure S9: Comparative analysis of effector gene counts among different fungal lifestyles
The distribution of effector gene counts across three fungal lifestyles: Endophyte, Plant Pathogen, and Saprobes. **Endophyte**: Represented by green, the effector gene counts for endophytes have an average value of 278.33. The box plot shows moderate variation with values clustering around the average. **Plant Pathogen**: Represented by red, plant pathogens exhibit an average effector gene count of 268.17. The distribution shows greater variability compared to endophytes, with several outliers indicating higher values. **Saprobe**: Represented by brown, saprobes have the lowest average effector gene count of 224.67. This group shows minimal variability in effector gene counts.

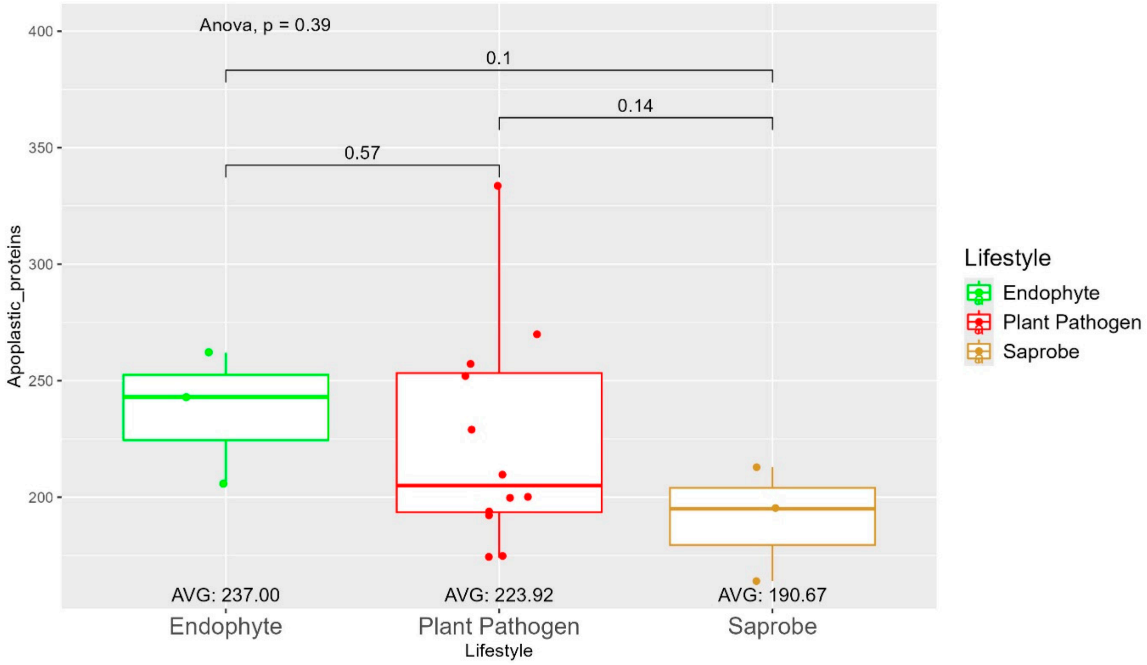

Figure S10: Comparative analysis of apoplastic protein counts among different fungal lifestyles
The distribution of apoplastic protein counts across three fungal lifestyles: Endophyte, Plant Pathogen, and Saprobes. **Endophyte**: Represented by green, the apoplastic protein counts for endophytes have an average value of 237.00. The box plot shows moderate variation with values clustering around the average. **Plant Pathogen**: Represented by red, plant pathogens exhibit an average apoplastic protein count of 223.92. The distribution shows greater variability compared to endophytes, with several outliers indicating higher values. **Saprobe**: Represented by brown, saprobes have the lowest average apoplastic protein count of 190.67. This group shows minimal variability in apoplastic protein counts.

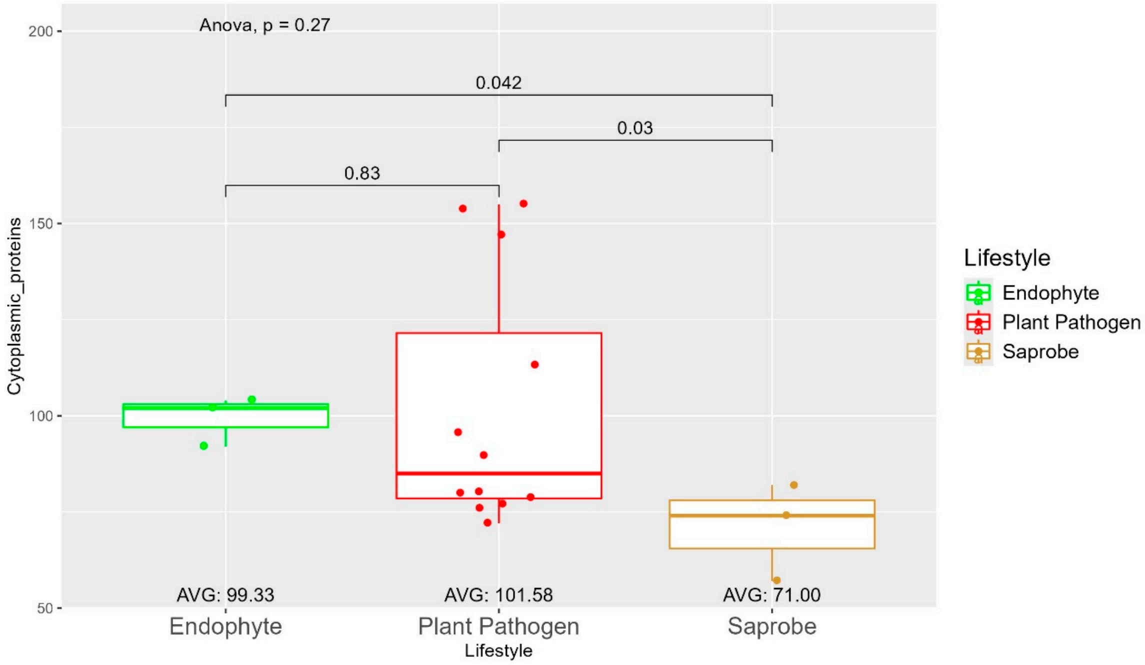

Figure S11: Comparative analysis of cytoplasmic protein counts among different fungal lifestyles
The distribution of cytoplasmic protein counts across three fungal lifestyles: Endophyte, Plant Pathogen, and Saprobes. **Endophyte**: Represented by green, the cytoplasmic protein counts for endophytes have an average value of 99.33. The box plot shows minimal variation with values clustering around the average. **Plant Pathogen**: Represented by red, plant pathogens exhibit an average cytoplasmic protein count of 101.58. The distribution shows greater variability compared to endophytes, with several outliers indicating higher values. **Saprobe**: Represented by brown, saprobes have the lowest average cytoplasmic protein count of 71.00. This group shows minimal variability in cytoplasmic protein counts.

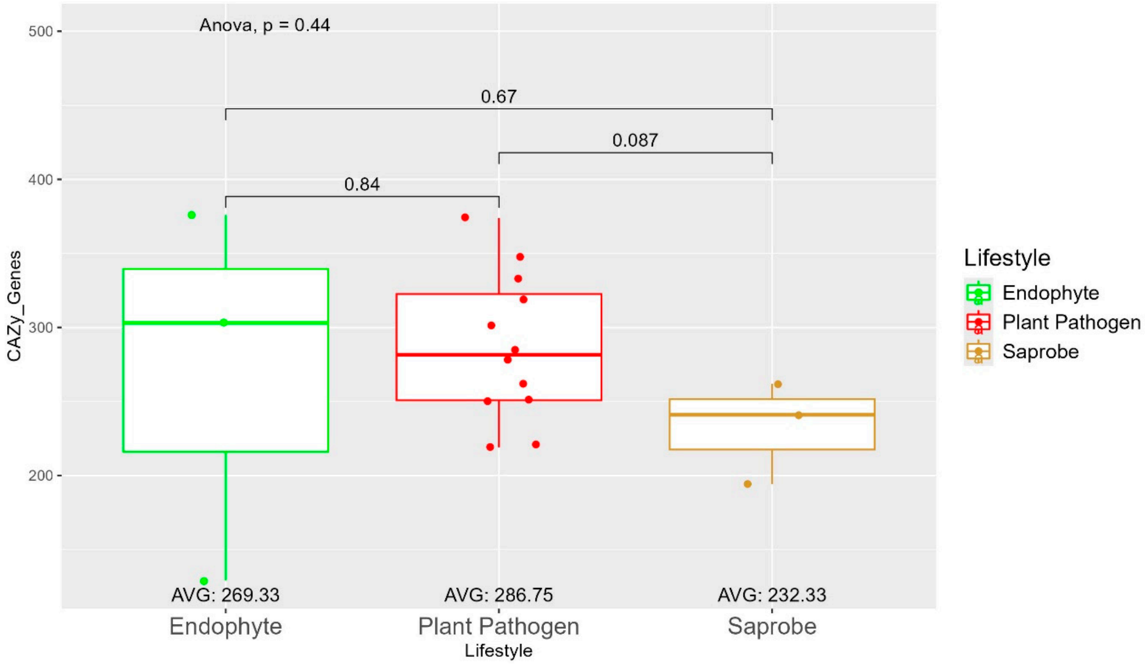

Figure S12: **Comparative analysis of CAZy gene counts among different fungal lifestyles**
The distribution of CAZy gene counts across three fungal lifestyles: Endophyte, Plant Pathogen, and Saprobe. Endophyte: Represented by green, the CAZy gene counts for endophytes have an average value of 269.33. The box plot shows a broad distribution of values, with some outliers indicating lower gene counts. Plant Pathogen: Represented by red, plant pathogens exhibit the highest average CAZy gene count of 286.75.

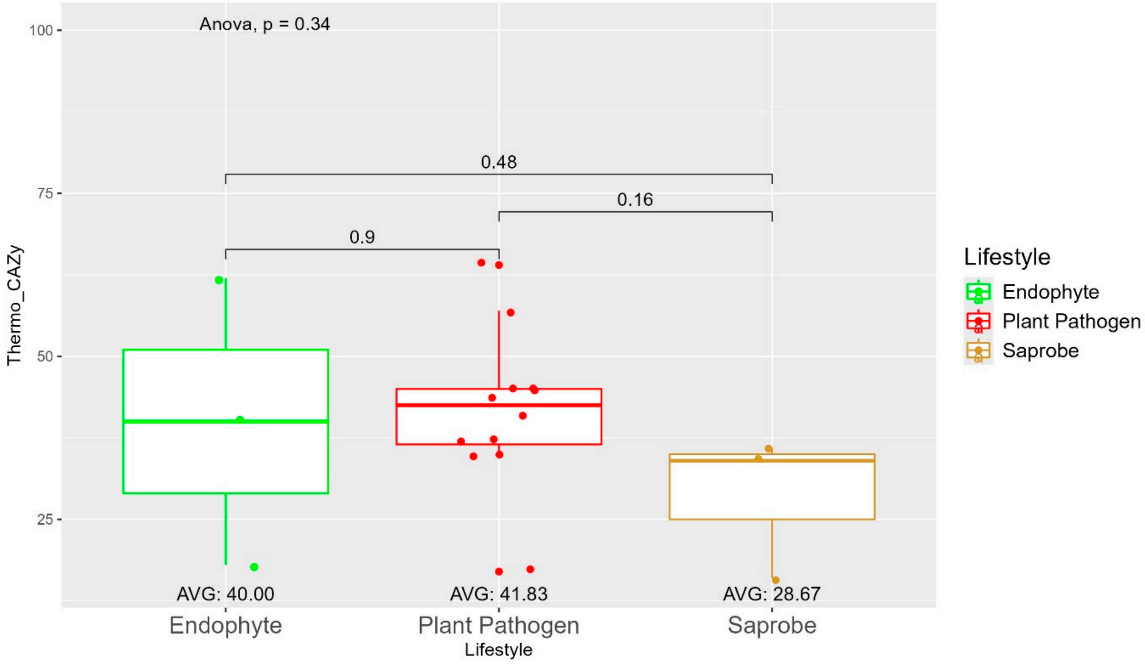

Figure S13: Comparative analysis of thermo-adapted CAZy gene counts among different fungal lifestyles.
The distribution of the thermo-adapted CAZy gene counts across three fungal lifestyles: endophyte, plant pathogen, and saprobe. Endophyte: Represented by green, the thermo-adapted CAZy gene counts for endophytes have an average value of 40.00. The box plot shows moderate variability, with values spread around the average. Plant Pathogen: Represented by red, plant pathogens exhibit a slightly higher average thermo-adapted CAZy gene count of 41.83. The distribution shows greater variability compared to endophytes, with a few outliers indicating higher values. Saprobe: Represented by brown, saprobes have the lowest average thermo-adapted CAZy gene count of 28.67, with moderate variability observed.

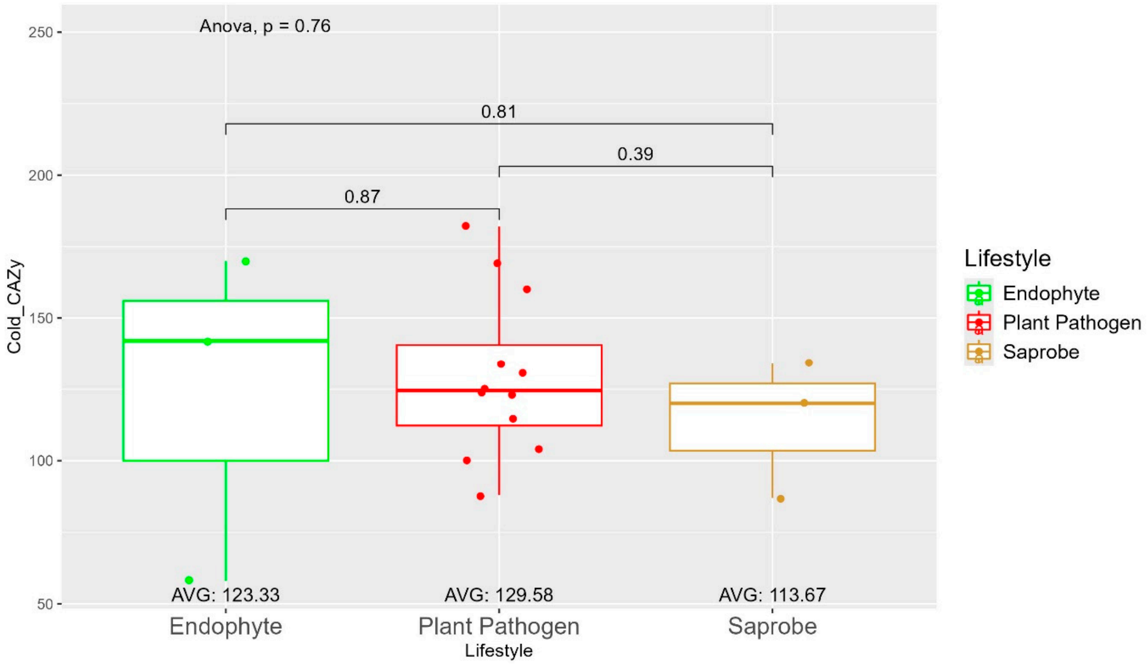

Figure S14: Comparative analysis of cold-adapted CAZy gene counts among different fungal lifestyles.
The distribution of cold-adapted CAZy gene counts across three fungal lifestyles: endophyte, plant pathogen, and saprobe. Endophyte: Represented by green, the Cold-adapted CAZy gene counts for endophytes have an average value of 123.33. The box plot shows a wide range of values, indicating significant variability. Plant Pathogen: Represented by red, plant pathogens have the highest average Cold-adapted CAZy gene count of 129.58. The distribution shows variability with several outliers indicating both lower and higher values. Saprobe: Represented by brown, saprobes exhibit the lowest average Cold-adapted CAZy gene count of 113.67, with the data showing a moderate range of variation.

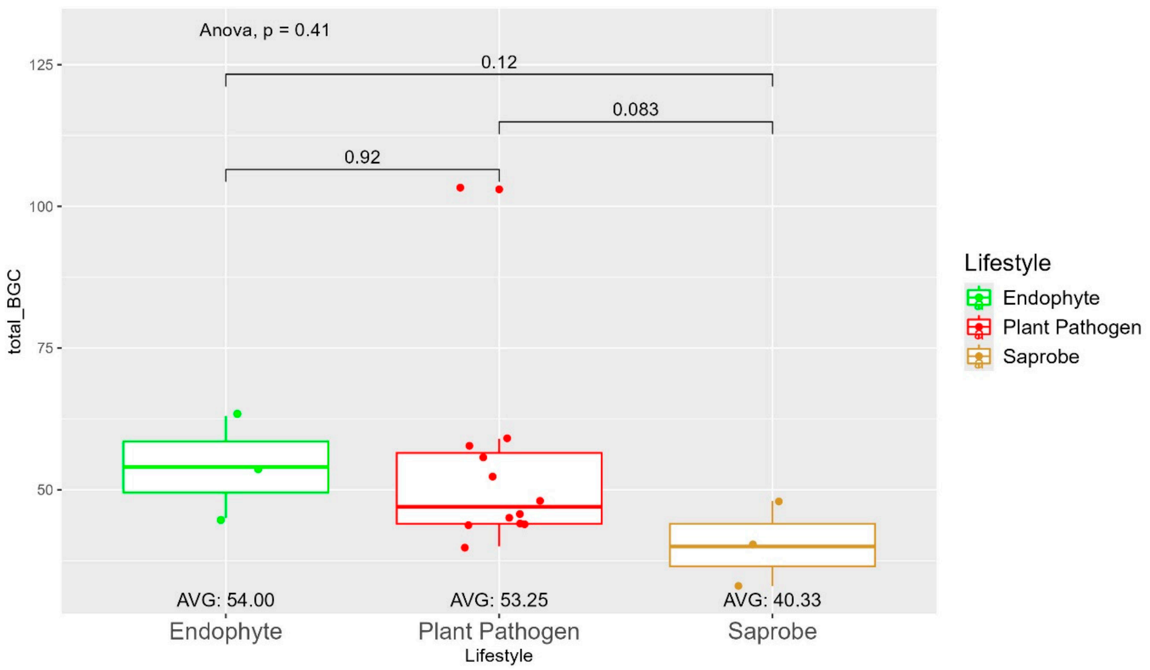

Figure S15: Comparative analysis of total BGC counts among different fungal lifestyles

The distribution of total biosynthetic gene cluster (BGC) counts across three fungal lifestyles: Endophyte, Plant Pathogen, and Saprobes. **Endophyte**: Represented by green, the total BGC counts for endophytes have an average value of 54.00. **Plant Pathogen**: Represented by red, plant pathogens exhibit an average total BGC count of 53.25. compared to endophytes. **Saprobe**: Represented by brown, saprobes have the lowest average total BGC count of 40.33.
